# Preparation and Application of Hybrid Nanomaterials

**DOI:** 10.3390/nano8110891

**Published:** 2018-11-01

**Authors:** Daniela Meroni, Silvia Ardizzone

**Affiliations:** 1 Dipartimento di Chimica, Università degli Studi di Milano, Via Golgi 19, 20133 Milano, Italy; 2 Consorzio Interuniversitario Nazionale per la Scienza e la Tecnologia dei Materiali (INSTM), Via Giusti 9, 50121 Firenze, Italy

The growing demand of new materials with tailored physicochemical properties has propelled hybrid materials to a position of prominence in materials science by virtue of their remarkable new properties and multifunctional nature. Hybrid nanomaterials, formed by two or more components connected at the nanometer scale, combine the intrinsic characteristics of its individual constituents to additional properties due to synergistic effects between the components [1,2]. As a result, the properties of hybrid nanomaterials can be tuned by changing their composition and morphology, leading to materials with enhanced performance characteristics, such as high thermal stability, mechanical strength, light emission, gas permeability, electron conductivity, and controlled wetting features [3,4]. Owing to their wide spectrum of accessible properties, hybrid materials are emerging platforms for applications in extremely diverse fields such as optics, microelectronics, smart coatings, health and diagnostics, photovoltaics, fuel cells, pollutant remediation, catalysis, and sensing [5,6,7,8]. This Special Issue, with a collection of 13 original contributions and two literature overviews, showcases some of the latest advances in this burgeoning and highly interdisciplinary research field, with the aim of highlighting potential applications in diverse fields, present challenges, and research outlooks.

Several articles in this Special Issue focus on the synthesis of materials or devices designed for pollution remediation. In the feature article by Liao et al. [9], hybrid surface coatings were prepared by modifying TiO_2_ films with Au nanoclusters by gas-phase beam deposition. The gold distribution onto the semiconductor support was highly homogeneous and provided efficient plasmonic photocatalytic activity. Tests of stearic acid degradation performed both under UV and green LED light showed a promoting effect due to the metal nanoclusters, especially under green light irradiation. The feature paper by Panzarasa and coauthors [10] presents a different approach to pollutant remediation, making use of natural renewable sources. Sepia melanin was used as an active component in hybrid adsorbent materials, owing to its ability to efficiently bind several organic compounds. The resulting hybrid material proved efficient, stable, easily recoverable and showed good reusability. Also in the work by Ren and coauthors [11], agro-alimentary waste is valorized as a starting material for hybrid material preparation. Magnetite-carbon nanocomposites were prepared by a hydrothermal procedure adopting pomelo peels as carbon source. The resulting hybrids were used as adsorbents to extract fungicide residues from homogenized fruit samples. One of the main issues in the pollutant remediation of surface waters and wastewaters by adsorption and/or degradation processes is represented by the removal of finely dispersed adsorbents/photocatalysts upon treatment. In the work by Lu and coauthors [12], a magnetic separation procedure is proposed to solve this problem: Hybrid magnetic iron oxides were deposited onto MoS_2_ nanosheets in the presence of metallic iron. By combining direct redox and Fenton processes, the hybrid provided simultaneous degradation of both toxic inorganic (Cr(VI)) and organic compounds (4-chlorophenol). Moreover, the nanocomposites could be separated magnetically from the treated effluent, showing good reusability.

In the last decade, hybrids based on carbon nanomaterials, such as carbon nanotubes, have raised a great deal of interest in several fields [13,14]. In the work by Das and coauthors [15], the in situ formation of either crystalline metals or metal oxides onto multiwalled carbon nanotubes (MWNT) was achieved by modifying the sol-gel conditions of the precipitation reaction, in the absence of any oxidizing or reducing agent, using the electrochemical potential as a control parameter. The reaction occurrence was made possible just by the surface energy and composition of the MWNT activated surfaces, which act as nucleation sites for the growth of the crystals. By the same principles, in the feature paper by Sansotera et al. [16], the successful functionalization of MWNT by perfluoropolyether chains was controlled by the surface features of the carbon nanotubes. The resulting covalent bond produced relevant modifications of the MWNT surface energy imparting superhydrophobic behavior; branched chains, bearing CF_3_ groups, produced a higher functionalization degree with respect to linear ones. The functionalization appeared to affect the pore size distribution of MWNT, mainly in the case of branched chains, while the conduction properties were only weakly modified. The control of the porosity and surface features of carbon materials is also the focus of the work by Lu and coauthors [17]. They proposed a controlled modification of mesoporous carbon by Mg and N in the presence of a non-ionic surfactant, giving rise to a higher microporosity and to two types of basic sites. Thanks to the enhanced morphological and surface features, the resulting materials showed increased CO_2_ adsorption, more than twice with respect to the pristine material.

Another field of applied science currently benefitting from hybrid materials is health care. Potential biomedical applications are envisaged in the works by Truong et al. [18] and by Predoi et al. [19]. Truong and coauthors [18] reported the synthesis of vertically aligned Cu-doped Zn nanorods grown on a platform of Cu_3_Si nanoblocks. The prepared nanocomposites showed an extended absorption edge and bioluminescence in the visible region, which paves the way to their application as bio-probes and luminescent markers. The work of Predoi et al. [19] deals with the very important topic of alternative antimicrobial agents for disinfection. Antibiotic resistance is becoming an increasingly major concern worldwide and has prompted the research of alternative treatments or medications. Predoi and coauthors [19] described the antimicrobial activity of essential oils deposited onto hydroxyapatite: Hydroxyapatite coated by lavender essential oil showed higher antibacterial activity with respect to other essential oil and, thanks to its biocompatibility, could be proposed to combat infections following prosthetic implantation. Regenerative medicine is also the topic of the review article by Batool et al. [20], more specifically, the new bioengineering approaches in terms of periodontal tissues and bone regeneration. The review sheds light on the use of bioactive hybrid scaffolds, such as functionalized membranes, for the controlled local delivery of anti-inflammatory drugs and growth factors for the treatment of periodontal diseases.

The Special Issue showcases a broad range of application areas of hybrid devices, including self-cleaning coatings [21], sensors [22], catalysis [23], optoelectronics [24], and photovoltaics [25]. The feature article by Vázquez-Velázquez et al. [21] presented covalently functionalized TiO_2_–SiO_2_ binary systems dispersed in an acrylic matrix, giving rise to hybrid films with excellent transparency and superhydrophilic properties. The authors discussed the synergistic effects in the nanocomposite on the grounds of the chemical interactions among the constituents and their morphology.

Wang and coauthors [22] reported a carefully designed hydrothermal synthesis giving rise to beautiful flower-like nanocomposites based on SnO_2_ nanorods and nano-sheet graphitic carbon nitride. The hybrid materials showed promising results as gas sensors for ethanol detection: The improved sensor response of the hybrids with respect to literature data, is discussed on the grounds of the band structure, resulting from the heterojunction between the two semiconductors, and of the increased number of gas adsorption sites.

The synthetic approach plays a key role also in the work by Jodłowski and coauthors [23] where the preparation of nanocomposites between zirconia and non-noble metal oxides, to be used as catalysts for methane combustion, was promoted by sonochemistry. The ultrasound treatment produced an optimal dispersion of the oxides onto the support leading to enhanced catalytic activity.

The communication by Kim et al. [24] presents a transparent and conductive hybrid material for use as transparent electrode in flexible electronics. Bidimensional silver nanowires deposited onto PET layers and decorated with nanometric Ti layers were proposed as a flexible substitute to conventional transparent conductive oxides, such as indium tin oxide (ITO). The titanium layer, deposited by electron-beam evaporation, imparted improved ambient-stability under high-temperature and high-humidity conditions and promoted a net increase in the electrical conductivity with respect to the pristine materials, yielding an 88% transparency rate and an electrical performance that is better than commercial transparent conductive electrodes.

The Special Issue is completed by a review article by Wu and coauthors [25] dealing with a very high profile topic in the energy conversion community: Perovskite-based solar cells (PSCs). Organic-inorganic perovskites have raised world-wide attention in recent years due to their unique electronic, optical and transport properties. In the last few years the power conversion efficiency of PSCs has increased explosively from 3.8% (2009) to about 22% (2017) [26]. However, the stability of perovskite solar cell devices is still unsatisfactory, particularly in the presence of moisture and light illumination. The role played by composition, structure and hybrid architectures were examined in detail in the review and the material design was proposed as a tool to control the material stability and conversion efficiency.

In summary, this Special Issue of Nanomaterials titled “Preparation and Application of Hybrid Nanomaterials” compiles a series of original research articles and review papers providing new insight on the preparation and on the wealth of applications of hybrid nanomaterials. We are confident that this Special Issue will provide the reader with an overall view of the latest prospects in this fast evolving and cross-disciplinary field.
